# Value and learning from carer involvement in a cluster randomised controlled trial and process evaluation - Organising Support for Carers of Stroke Survivors (OSCARSS)

**DOI:** 10.1186/s40900-020-00193-7

**Published:** 2020-05-08

**Authors:** C. Mitchell, K. Burke, N. Halford, K. Rothwell, S. Darley, K. Woodward-Nutt, A. Bowen, E. Patchwood

**Affiliations:** 1 Faculty of Biology, Medicine and Health, University of Manchester, Manchester Academic Health Science Centre, Manchester, UK; 2 Patient and Carer Public Involvement (PCPI) contributors: lay members of the OSCARSS Carer Research User Group, Manchester, UK; 3 Stroke Association support services, Stroke Association, London, UK; 4 Research & Innovation, Northern Care Alliance NHS Group, Salford, UK

**Keywords:** Patient, Carer, Public involvement (PCPI), Cluster randomised controlled trial, Co-design, Caregiver, Carer, Stroke, Decision-making

## Abstract

**Background:**

Patient, Carer and Public Involvement (PCPI) should be embedded in health care research. Delivering PCPI can be challenging, but even when PCPI is carried out it is rarely reported resulting in lost opportunities for learning. This paper aims to describe PCPI in the OSCARSS study, a pragmatic-cluster randomised controlled trial with an embedded economic and process evaluation.

**Methods:**

A carer research user group (RUG) co-developed OSCARSS to evaluate how to best deliver support to caregivers of stroke survivors. The PCPI activity involved regular meetings and preparatory work, from the initial conceptualisation of the study through to dissemination. Written reports, structured group discussions and individual interviews were carried out with the RUG and researchers to capture the added value and learning. This paper was co-authored by two of the RUG members with contributions from the wider RUG and researchers.

**Results:**

The core six members of the caregiver RUG attended the majority of the meetings alongside three researchers, one of whom was the co-chief investigator. PCPI was instrumental in changing many aspects of the research protocol, design and delivery and contributed to dissemination and sharing of good practice. There were challenges due to the emotional toll when PCPI members shared their stories and the extensive time commitment. Positive experiences of learning and fulfilment were reported by the individual researchers and PCPI members. Wider organisational administrative and financial support facilitated the PCPI. The researchers’ existing positive regard for PCPI and the clear focus of the group were key to the successful co-design of this research.

**Conclusions:**

The value and learning from the PCPI collaborative work with the researchers was of benefit to the study and the individuals involved. Specific PCPI influences were a challenge to pinpoint as successful co-design meant the researchers’ and carers’ contributions were intertwined and decision-making shared.

## Plain English summary

Patient, Carer and Public Involvement (PCPI) is a fundamental part of health research but is rarely documented thoroughly in study reports. This collaboratively written paper, describes the process and impact of a group of caregivers working within a health research study. This carer Research User Group (RUG) worked in partnership with researchers and Stroke Association (a national charity supporting stroke survivors and their families) on the Organising Support for Carers of Stroke Survivors (OSCARSS) study. The collaboration was developed within nationally recognised good practice guidance.

OSCARSS investigated how to best support caregivers of stroke survivors. Informal caregivers make a valuable contribution to the health and wellbeing of individuals and the communities in which they live, sometimes to the detriment of the caregiver’s own physical health, family and social networks. Although supporting caregivers is recognised as a national priority, there is little guidance on how best to do this. Involving a RUG, as active partners during the study, helped to gain a better understanding of the varying needs and experiences of caregivers. The regular exchange of ideas and contributions from a range of caregivers helped make the study truly collaborative. The RUG’s perspectives helped the research team to adjust their approaches to some of the study processes and interpret findings. Participation could be emotionally triggering for the caregivers and the time commitment was substantial but overall, the caregiver RUG reported positive experiences and learning as individuals.

## Background

This co-authored paper reports how a research user group (RUG), of people with experience of caregiving and stroke, worked with researchers to develop and support a large mixed-methods study called Organising Support for Carers of Stroke Survivors (OSCARSS), including a cluster randomised controlled trial [1]. We chose to write this paper in the third person when referring to ourselves, the RUG and the researchers. The collaborative approach to working with the RUG adhered to the framework for collaborative Patient, Carer, Public Involvement (PCPI) as outlined by the NHS and NIHR [2–5]. PCPI is not always fully embedded in research due to perceptions of the many challenges involved; from organisational support, to funding, to practical difficulties in recruiting interested contributors and a lack of training or skills for both researchers and PCPI participants [6, 7]. Few research studies report, or even acknowledge, the involvement of PCPI [8] perhaps because it often only exists at a tokenistic level and is therefore constrained in the value it could add. This lack of reporting hinders initiatives to increase PCPI in health research [9–11].

The OSCARSS RUG collaborated on this study, looking at how best to support the caregivers of stroke survivors working in partnership with a National stroke charity, the Stroke Association. Many stroke survivors are left with a range of disabilities [12] resulting in a loss of independence and a need for care from partners and family members [13, 14]. Caregivers take on responsibility that can affect their own physical health, family and social networks as well as emotional well-being [15–18], while providing care with a high financial value to the health and social care service [19, 20]. Supporting caregivers is a high priority nationally [21] but reviews of the evidence suggest that is not clear how best to do this [22–24].

The involvement of the RUG in OSCARSS followed the guiding principle of patient and public involvement of “nothing about me without me” [25]. Our intention is to describe the PCPI process in this study, the learning from this and the value of involvement in terms of both the product (the research protocol and findings) and the producers (the individuals involved) [26]. It has been suggested that PCPI should be qualitatively, rather than quantitatively evaluated to reflect the changing and evolving nature of PCPI in terms of describing the relationships, social interactions and networks developed through PCPI [27, 28]. In this paper we attempted to use the consensus Guidance for Reporting Involvement of Patients and the Public (GRIPP2) [29]. Our main objective is to show the RUG members’ and researchers’ story, to reflect how involvement made a difference and how the PCPI shaped learning, changed thinking and enhanced the research [28].

### Description of the study

The OSCARSS protocol has been published [1] and the results are described elsewhere (Patchwood E, Woodward-Nutt K, Rhodes S, Batistatou E, Camacho E, Knowles S, Darley S, Grande G, Ewing G, Bowen A: Organising support for Carers of stroke survivors (OSCARSS): a cluster-randomised controlled trial with economic evaluation, submitted; Darley S, Knowles S, Woodward-Nutt K, Mitchell C, Grande G, Ewing G, Rhodes S, Bowen A, Patchwood E: Challenges implementing a carer support intervention within a national stroke organisation: findings from the process evaluation of the OSCARSS trial, submitted). OSCARSS aimed to investigate the clinical and cost-effectiveness of the adapted Caregiver Support Needs Assessment Tool (CSNAT) for carers of stroke survivors [30]. OSCARSS was a longitudinal, multi-site, pragmatic, cluster randomised controlled trial with an economic and embedded process evaluation. We compared intervention to a control of standard carer support practices and our primary outcome was caregiver strain, with secondary outcomes including distress, positive appraisals of caregiving, mood and health impact. The study ran for 33 months, randomised 35 clusters (Stroke Association services) across England and Northern Ireland who recruited 414 caregivers. An additional 170 staff members provided data for the OSCARSS process evaluation.

### Aim of PCPI

The aim of the RUG was to ensure the study produced valid evidence, relevant to the caregivers of stroke survivors.

## Methods

To set up the RUG the researchers leading the OSCARSS study contacted relevant local patient and carer groups and individuals interested in being contacted about research, to invite them to an open meeting to discuss the proposed research. Twenty six potential carers were approached. There were no selection criteria other than experience of stroke and caring.

Ten individuals initially joined the RUG, four of whom dropped out. Of the six long-term members, four were consistent members from the start (December 2015) until the final RUG meeting (August 2019), including the two named co-authors KB and NH. A fifth started at a slightly later date but continued to the end, and the sixth started as a member but struggled to attend meetings during the final year of the study. Of the 10 involved at the start there were seven women and three men with an age range of approximately 34 years to 76 years. Eight of the RUG were caring for partners and two for a parent (stroke survivors) with one a stroke survivor who was also a carer. The stroke survivors they cared for were between 3 and 5 years post-stroke. Three of the six long term members were men.

The researchers taking the lead in PCPI included the co-chief investigator (EP) and the project manager (KWN). Both researchers had prior experience of PCPI in research, and both had professional experience of working with stroke survivors and their families in clinical and research settings. The other co-chief investigator [AB] was supportive of PCPI and attended several meetings. Most meetings were attended by three researchers, including administrative support who helped set up meetings, organised financial matters and took notes.

The RUG met monthly (3 h meetings including lunch) at the start of the project when workload was high, then once every two months when recruitment to the study had started, then quarterly as the workload reduced, with a final meeting in August 2019. Between meetings RUG members were asked to read information and prepare for meetings. RUG members were compensated for preparation and meeting time at the rate of £10 per hour, paid in vouchers and reimbursed for out of pocket expenses in cash. Meetings were held, with no charge, in a local charity-run facility in a community setting, convenient and accessible to all including wheelchair users. Additional support and extra pre-meeting preparation time was given to support any member with specific learning needs. Refreshments and lunch were provided and dietary needs catered for. A collaborative approach was taken in facilitating the meetings and everyone contributed to the agenda. The RUG and researchers agreed their terms of reference: everyone is equal; everything is confidential; take turns; no interruptions; give each person time; members show respect and support; discussions and decisions are written down by a researcher to be circulated after meetings; anyone can drop out if they wish.

Activities of the RUG included: adapting the protocol, intervention, outcome measures; communicating updates to all involved via newsletters; ensuring pertinent recruitment strategies and follow-up; evaluation of the study conduct, interpreting and dissemination of findings. Proposed meetings were flexible according to PCPI and study need but ensuring regularity of contact throughout the process. A caregiver, who was not involved in the RUG, was identified to sit on the Trial Steering Committee as an independent lay member. The RUG could contact the chair of the Trial Steering Committee at any point during the study independently of the research team.

### Data and analysis

Early in the study the RUG decided to capture and share data on their reflections during OSCARSS to describe the value and learning of PCPI. Data from written documents and team discussions / interviews (see below) provided rich descriptions of the experiences and perceptions of the RUG members and researchers about their involvement in this study.

Written documents included meeting notes, which were circulated to the RUG to be checked, and financial records of PCPI activity costs (not including research and administrative staff time). In addition, the RUG decided to document their experiences in a written dossier to encourage contributions from less vocal members of the group, with the headings: background; composition of the group; individual reasons for getting involved; highlights and achievements and what the RUG brought to the research.

In addition, structured discussions were conducted with all members of the RUG (group discussions and one-to-one) and, independently, with two researchers to understand: i) the reasons for RUG engagement in research, ii) the reasons for researchers engaging the RUG in OSCARSS, iii) the value and learning from the RUG and how this influenced the study itself, and iv) the benefits and challenges of PCPI involvement for both researchers and the RUG. These discussions were audio recorded, with consent and ethical approval and transcribed. The first author (CM), involved in the process evaluation interviews but no other aspect of the OSCARSS study, conducted a thematic analysis [31]. CM initially coded all interviews and actively derived themes based on the structured discussions and study aims to understand reasons for engagement; value and learning; benefits and challenges. These themes and their meaning were discussed with the carer co-authors, KB and NH in order to ensure consensus and shared meaning. Direct quotes were selected below where they illustrated specific themes; with quotes anonymised using a system of R (respondent) and each respondent numbered according to when they first spoke during interview. The RUG were numbered R1 to R5 and the researchers interviewed separately were R6 and R7.

## Results

This section presents the main themes from the data, the impact and the financial cost of PCPI.

### Motivation for PCPI

The majority of RUG members were motivated by altruism and chose to get involved with the aim that other carers would benefit from their experiences:

*“Hopefully, trying to represent other carers and enable the caring process to make it a bit easier for everybody else, the same as that we've all had stumbling blocks along the way and barriers that we've come across where we haven't been able to do this or we've not had the right information. So, you know, hopefully we can enable other people to then go forward and have the process a bit easier”* R3

There were some indicators from the RUG that they also hoped for personal benefits, such as establishing a support network with other carers. The RUG members at the end of the study had a clear idea of PCPI in research and reported that research about a certain population should include those affected to provide an authentic voice and to ensure the research included what was important to those individuals. The RUG had a clear sense of levels of involvement and awareness of more tokenistic engagement that sometimes occurs but reported feeling fully involved in OSCARSS and working in partnership with the researchers. There was certainly a feeling that researchers who had theoretical knowledge needed to understand what was important to those affected and this was a key part of the carers’ role:

*“Yeah, stay away from the textbooks. And actually, speak to people that are dealing with the subject that you are researching on.”* R5

The RUG expressed their view of the reciprocal nature of their role. They understood that ‘researchers’ in general could view PCPI as a hindrance but suggested that PCPI would ultimately enable the researchers to produce higher quality research:

“*But at the end of the day, it's their name on the research, so they want to get the best out of it. When they put the papers up and present that, it's their name on the bottom of that, so they want it to be the best it can be. So, you know, it's them that's going to get the glory out of it, so they need to be able to do it the best they can. And if they feel that this works, then it doesn't matter how many times we change it, does it, as long as it gives the best evidence*” R1

### Motivation for researcher PCPI

The researchers taking the lead in RUG carer group engagement were the co-chief investigator (EP) and the project manager (KWN) and they felt well supported by the other co-chief investigator (AB). They had strong beliefs of the importance of PCPI in research, of involving those with lived experience of the topic under investigation at earliest possible moment in health research:

*“We need to do health economics, so we would get a health economist because I don’t know about health economics, so we wanted to develop something to help carers and I don’t know about caring so let’s talk to carers, it’s so self-evident” R6*

### Value of PCPI to OSCARSS

The RUG reported a real sense of being involved in developing the research as equal partners with trust and belief between themselves and the researchers. This trust came from the researchers taking action when they said they would or explaining what barriers they faced if they couldn’t:

*“for me, that gives it a sort of added dimension in a way that, like you say, it's not tokenism, it's actually there's been a process and a long term process where there's a relationship of trust, a relationship of understanding”R2*

The RUG described having a sense that they had really made a difference to this research and a real pride in the work they carried out, and this explains the long term commitment that many of the RUG had staying involved throughout this study. Some planned to get involved for a few months and ended up staying involved for several years:

*“I think initially if they said, you know, are you going to commit to this group for, like, four years or nearly four years, we'd all go no”* R3

One of the key messages that came out of the RUG was that they felt a strength of the group was its diversity. They reported that there were people from a range of socio-economic backgrounds, ages, different carer relationships, different levels of education and job backgrounds and this helped them to offer a variety of perspectives. They did suggest that the inclusion of people from other cultural backgrounds could have enhanced the group to give another viewpoint:

*“Because some cultures in this country are very community-orientated and some cultures are very isolated within each other. So whereby you will find a whole community will get behind one person to help them, the next person will be left completely to their own devices while everyone stands back and watches” R5*

The researchers described the power of the PCPI input which they feel gives credibility to the research:

*“There is huge leverage that the carers’ voice has … and we could use that as people really listen if the carers say this is really important. It just seems to elevate people’s respect for the project” R6*

The opinion of the RUG was of real value when dealing with the potential challenge of participant recruitment when service providers act as ‘gatekeepers’, making judgements as to whether someone is invited to be a potential participant. Being able to confirm to those involved in recruitment that the RUG felt it was essential that potential participants were offered the choice, regardless of recruiters’ opinions, was helpful:

*“ … and this was particularly the case when trying to change staff behaviour, when we said that our carer group felt [carers] should make that decision and people should be asked at this point” R7*

PCPI activity and the value to the study is summarised in Table 1.

**Table 1:** RUG activity log

RUG activity	Value to OSCARSS
Adaptation of the chosen intervention (CSNAT) (within copyright boundaries)	Focussed on stroke rather than palliative care (CSNAT-Stroke) and developed a paper action plan
Development of staff training package to implement intervention	Improved likelihood of intervention fidelity and increased staff ‘buy-in’ to the approach
Early protocol discussion raised the possibility of carers not identifying as ‘carers’ which may affect recruitment	Consideration of the use of the term ‘carer’ and highlighting awareness of this in recruitment
Participant recruitment: RUG gave advice on possible experiences of carers and suggested ways to approach recruitment to encourage engagement. RUG suggested the first contact with the carer participants should be in person or over the phone to increase recruitment	• The protocol was changed so that potential participants were given courtesy calls in advance of postal packs being sent out. • Recruitment protocol amended so that potential participants were given more time to decide and could be approached later, this was anecdotally reported to have increased recruitment rates
Selection of the primary outcome from a choice of measures	A primary outcome selected that was deemed relevant and important to carers, with language that was accessible and low perceived burden for completion
Developing the participant information sheet	Accessible and jargon-free for carer participants - used as an example for other research
Promotion of the role of the RUG in this study at conferences	Opportunities for the RUG to develop their own skills and knowledge around dissemination
Interpretation and dissemination of the study process and findings.	• Actively involved in interpreting qualitative themes in the process evaluation and interpretation of study findings • Wrote an accessible and jargon-free report on findings for carer participants • Presented work at conferences internationally and nationally
Publishing the experience of PCPI involvement in the OSCARSS study	The RUG wanted to tell others about the importance of getting involved in research and the two co-authors have been actively involved in this paper

### Personal impact

The RUG said they felt valued and listened to during their work on this study and this gave them a sense of real worth:

*“We actually feel that we’ve been listened to rather than it’s gone through three or four different people, edited by each one and then there’s a resemblance of something that you’ve said. So, like I say you feel more valued”R5*

The opportunities for many of the RUG to get involved in teaching at the University, running workshops and presentations at national and international conferences also reinforced this sense of having something to offer and feeling valued. Many of the RUG members found personal support from their membership of a group where others understood the pressures of being a carer and supported each other to understand the research process.

RUG members reported that occasional frustrations were related to the fact that research had to be done in a certain way, within the confines of the protocol and this could limit the changes they wished to make. For example, they had suggested further changes to the intervention tool, CSNAT, that could not be implemented due to copyright restrictions. The complexity of the research and the amount of information was challenging and could be overwhelming, but they report being well supported by the researchers in reducing jargon.

Some of the personal challenges related to the initial stages of getting to know the other members of the RUG, e.g. “introductions” meant describing their own personal circumstances. In some cases, members found this upsetting as they had to ‘re-live’ their story. Some of the members describe they have learnt to listen to others better and think about other peoples’ perspectives:

*“I’ve realised that no, it might actually be the way that I’m thinking about it that’s the wrong bit, not the way that they’ve explained it. So, finding the common ground there; like I say, it’s just been a lot of life lessons for me as well”R5*

The group reported that getting to know each other took time, everyone had a different style of interacting, some people liked to think and reflect and other members were more vocal. Despite the level of time commitment, RUG members always felt this was optional and any activities they were invited to participate in were always offered with no sense of expectation. The time commitment included the preparation before meetings, the meetings and the work outside of the meetings reading minutes and research documents as well as dissemination activities attending conferences.

The researchers involved approached the RUG with a positive view and a sense of empowering a group of people to have their voices heard. Researchers were conscious of the group dynamics and their potential position of power, which they attempted to offset by sharing their own personal experiences and understanding of caring.

### Challenges

The researchers, with their embedded prior belief of the value of PCPI, felt that the benefits of this co-produced research with the RUG outweighed the challenges. However the researchers felt it was important to consider the personal challenges to their involvement. Managing group dynamics could be difficult, where people worked through information at different speeds which led to frustrations or managing individual styles if people were more or less vocal in the groups. Building the trust of the group, sharing difficult information and managing the emotions of all those involved in this process could be both draining and emotionally challenging particularly at the start when people shared their experiences:

*“There were a lot of tears actually in that first meeting … it was very emotional in the first two meetings”R6*

In terms of organisational challenges the researchers had access to funding for this PCPI. The total cost of PCPI reimbursement for time, mileage, catering, conference and other dissemination events but excluding the costs of staff time, came to a total of £5000. Staff costs were not included here because the project was funded within a major applied health research programme with an understanding that staff could take whatever time was needed to work with the RUG (https://clahrcprojects.co.uk/resources/clahrc/greater-manchester). This meant there were two or three research staff facilitating the meetings and sending out preparatory work which helped to support the group dynamics, responding to those needing more explanation or support:

*“ … there were typically three of us in meetings, that’s very resource heavy isn’t it? One was taking notes, there might be two of us to manage communication and energies in the room … having the relationship between the people facilitating and the group was probably quite key to our success.”* R6

## Discussion

This paper described the PCPI in OSCARSS, a study that included a national cluster randomised controlled trial. Positive experiences were found by both the informal carer PCPI contributors and the stroke research team members who shared a belief that this partnership added value to the study. This successful outcome is likely due to the interplay of several factors: the altruism of the RUG members; the mind-set of the research team; financial and administrative resources to support PCPI; and partnership working with the Stroke Association (an organisation that represents stroke survivors, their families and friends). The long-term commitment of the RUG was sustained by developing close working relationships and trust between members and researchers. The researchers’ individual attitudes, and wider organisation support and funding from the broader research programme, allowed them to invest time building relationships and fully engaging in co-production. The successful PCPI during this study is described in this paper in terms of ‘value and learning’ as well as ‘how’ it was carried out with practical recommendations from the researchers and PCPI members to enthuse others interested in co-producing research [32].

Our PCPI process was consistent with existing guidance and recommendations [2, 5, 33]. Our findings in many ways reflect the methods reported by others for effective PCPI work, particularly the RAPPORT study [34] which suggests six actions for effective PCPI; clear purpose, role and structure; diversity; whole research team engagement; mutual understanding and trust between PCPI and researchers; opportunities for PCPI; reflecting on appraising and evaluating PCPI. We contribute to this evidence by describing how these actions were carried out during a large cluster randomised controlled trial where reporting PCPI is scarce [8] and the value of PCPI to our research [26]. We have attempted to report on the process and context of PCPI in line with reporting recommendations and comply with as many as possible of the items on the GRIPP2 reporting checklist [29].

Our findings show that the RUG were initially motivated to support OSCARSS through a wish to improve experiences for their peers. Their continued involvement through the four years from preparation, data collecting and analysis, through to dissemination, was a true sense of partnership and feeling valued. This reinforces the findings that having a clear purpose and research team engagement promote successful PCPI [34]. The broad research context of OSCARSS enabled the researchers to engage in true partnership working, where the programmatic and partnership funding allowed carers to develop the research at the earliest opportunity. The wider context of administrative support to organise finances, refreshments, room bookings and resources for two to three researchers at any one time to facilitate all meetings was considered a crucial factor in the success of this group. The researchers had time to prepare for meetings and to ensure the RUG were well prepared with a clear focus and structure, and had the opportunity within meetings to focus on building relationships [35, 36].

The wider organisational context supported the positive experience of PCPI in this study but the individual skills and experiences of the researchers involved should also be considered [11, 28]. The researchers had clinical and previous professional experience of PCPI work, they were open to all RUG suggestions and worked collaboratively, they demonstrated an ethos that PCPI work was fundamental to the study, which is not always the case with health researchers [37, 38] and is likely to have had a positive impact. It also seemed relevant that the co-chief investigator led the RUG, and this lent importance to the work of the PCPI reinforcing the value placed on their input. Other research indicates PCPI work is often the job of more junior members of the research team [11]. The RUG showed understanding of how they could be seen by some researchers as being problematic or challenging, but tokenism would not have sustained their interest or motivation. They shared some really useful insights into the power balance. The researchers needed PCPI for credibility but the RUG felt their input would improve the quality of the research. We hope this will dispel concerns other researchers have raised about how PCPI could be detrimental to researchers’ careers [11].

We did not quantitatively ‘evaluate’ or ‘measure’ PCPI involvement [1] as reflected in the GRIPP 2 checklist [29] but we do describe the value the RUG added to the OSCARSS study. It may be that RUG suggestions and ideas could have been ‘measured’, for example when we describe in Table 1 that giving carers longer to consider the study was anecdotally reported to have improved recruitment. However, the research team felt that truly, co-produced research where the PCPI members and researchers worked together can make it impossible to differentiate between the researchers and PCPI contributions and ‘measuring’ becomes complex. There was an overwhelming sense from the RUG about the benefits they brought to the research and they were clear in their view that they had improved OSCARSS; they felt the protocol and study management were enhanced by their involvement. The credibility of the ‘voice’ of the RUG is considered to be of great value to the researchers leading the study, and it may be that this benefit to health researchers is under-valued and difficult to measure quantitatively [11].

Inevitably there were challenges for the RUG members and researchers during this complex process of working together. There is considerable similarity e.g. both reported that time commitment was a challenge. Both recognised the issues around group dynamics, within groups and between the researchers and RUG members and particularly how this could be emotionally hard for everyone. Both researchers and RUG members acknowledged the power imbalance, and researchers made a conscious effort to involve RUG members throughout the research process. The RUG members described feeling that their role might be seen as making life difficult for ‘researchers’ in general and slowing the process down, but felt their job was to make the research relevant and ‘better’. Having a clear sense of purpose and opportunities to get involved in all aspects of the process help overcome some of these challenges and support effective PCPI [34]. Many of these challenges could not perhaps be foreseen or overcome. They were viewed as an accepted necessity of working with a broader range of individuals with their own unique contributions and stories of caring, as well as different expectations and external demands. In addition to previous recommendations for successful PCPI [33, 34, 39, 40] we suggest that financial support for very early PCPI can support partnership working.

## Conclusions

The OSCARSS study had embedded PCPI involvement and the RUG contributed to every stage of the study providing value and learning for the study, the researchers and the RUG members themselves. The positive impact of the RUG in OSCARSS and the work to report how it was done and how it changed the research allows us to contribute to the evidence base and offers some practical recommendations from the RUG and researchers to improve effective PCPI for other research teams. This description of the PCPI during OSCARSS is not intended to be an evaluation or a measure of impact but to provide a rich contextual overview of the development of the RUG and how the relationships, social interactions and networks allowed the RUG to contribute to and co-produce this complex study. This may support others embarking on PCPI.

## Data Availability

The data sets generated from interview do not have permissions to be shared beyond the research team.

## Abbreviations

OSCARSS: Organising Support for Carers of Stroke Survivors
PCPI: Patient, Carer and Public Involvement
RUG: Research User Group
GRIPP2: Guidance for Reporting Involvement of Patients and the Public
NIHR: National Institute of Health Research
CLAHRC: GM Collaboration for Leadership in Applied Health Research and Care Greater Manchester
